# Porcine Pluripotent Stem Cells Derived from IVF Embryos Contribute to Chimeric Development *In Vivo*

**DOI:** 10.1371/journal.pone.0151737

**Published:** 2016-03-18

**Authors:** Binghua Xue, Yan Li, Yilong He, Renyue Wei, Ruizhen Sun, Zhi Yin, Gerelchimeg Bou, Zhonghua Liu

**Affiliations:** 1 College of Life Science, Northeast Agricultural University, Harbin, 150030, China; 2 Department of Histology and Embryology, Harbin Medical University, Harbin, 150081, China; University of Texas at Austin Dell Medical School, UNITED STATES

## Abstract

Although the pig is considered an important model of human disease and an ideal animal for the preclinical testing of cell transplantation, the utility of this model has been hampered by a lack of genuine porcine embryonic stem cells. Here, we derived a porcine pluripotent stem cell (pPSC) line from day 5.5 blastocysts in a newly developed culture system based on MXV medium and a 5% oxygen atmosphere. The pPSCs had been passaged more than 75 times over two years, and the morphology of the colony was similar to that of human embryonic stem cells. Characterization and assessment showed that the pPSCs were alkaline phosphatase (AKP) positive, possessed normal karyotypes and expressed classic pluripotent markers, including OCT4, SOX2 and NANOG. *In vitro* differentiation through embryonic body formation and *in vivo* differentiation via teratoma formation in nude mice demonstrated that the pPSCs could differentiate into cells of the three germ layers. The pPSCs transfected with fuw-DsRed (pPSC-FDs) could be passaged with a stable expression of both DsRed and pluripotent markers. Notably, when pPSC-FDs were used as donor cells for somatic nuclear transfer, 11.52% of the reconstructed embryos developed into blastocysts, which was not significantly different from that of the reconstructed embryos derived from porcine embryonic fibroblasts. When pPSC-FDs were injected into day 4.5 blastocysts, they became involved in the *in vitro* embryonic development and contributed to the viscera of foetuses at day 50 of pregnancy as well as the developed placenta after the chimeric blastocysts were transferred into recipients. These findings indicated that the pPSCs were porcine pluripotent cells; that this would be a useful cell line for porcine genetic engineering and a valuable cell line for clarifying the molecular mechanism of pluripotency regulation in pigs.

## Introduction

The pig is an important farm animal and a useful experimental model for human disease due to its obvious physiological and immunological similarity with humans [1–3]. The pig also holds great potential for testing the safety of clinical stem cell transfer and related techniques. Embryonic stem cells (ESCs) have offered a wide range of cellular resources for developmental research and clinical applications. However, the encountered difficulty with genuine porcine embryonic stem cells (porcine ESCs) has greatly hampered the progress in these fields[3].

Efforts have been made on establishing porcine ESCs since the first group of reports about porcine ESC-like cell lines in 1990 [4–8], but no bona fide embryonic stem cell (ESC) lines that could fulfil all the characterization demands that mouse ESCs do have been established in pigs in past decades. Age, the source of embryos[9–12], the isolation methods of the inner cell mass (ICM) [13, 14], different feeder layers[6, 14–19], components in the culture medium, self-renewal-related cytokines, especially[15, 17, 18, 20, 21], and the atmospheric conditions[22] had been widely studied. However, the limited proliferation potency of most of the established porcine ESC-like cell lines prevented thorough characterization, except for the characterization of morphology and a few pluripotency-related markers, such as AKP, OCT4 and SOX2. This situation becomes even more complicated when the lack of validated antibodies and other related testing techniques is considered.

The culture system has been considered one of the most important factors for establishing a porcine ESC-like cell line. The facts that outgrowths and AKP-positive colonies could be isolated and cultured from porcine pre-implantation embryos indicated that there were pluripotent cells in the porcine embryos. However, the culture medium, most of which was modified from mouse ESCs or human ESCs culture medium, could not provide an effective environment for maintaining the self-renewal and proliferation of these putative porcine pluripotent cells, as it does for mouse ESCs and human ESCs establishment[23–25]. LIF and bFGF are the most important cytokines in the culture medium for maintaining the pluripotency of mouse ESCs [26] and human ESCs, respectively [27–30]. Although there have been reports showing that there is no LIF receptor in porcine ICM cells [31, 32], studies on porcine pluripotent cell lines have shown that the porcine pluripotent signalling pathway might depend on both LIF and bFGF [25, 32]. Therefore, the signalling pathway that regulates porcine pluripotency is still an open scientific question.

To obtain porcine pluripotent stem cell lines from early embryos, provide an opportunity to clarify the molecular mechanism of porcine pluripotency regulation, and obtain materials for porcine genetic engineering, we used *in vitro fertilization* (IVF) blastocysts as an embryo resource, which had advantages in the selection of precise embryo development stages for seeding; we also developed a new culture medium named MXV containing both hLIF and bFGF as a basic culture system in an atmosphere of 5% oxygen. AKP-positive colonies with human ESCs morphology formed after seeding day 5.5 blastocysts, and these colonies could be passaged more than 75 times over two years. The characterization of the named porcine pluripotent stem cells (pPSCs) showed that they are pluripotent cells that could contribute to the viscera and placenta development of chimaera foetuses.

## Materials and Methods

### Animals

Porcine ovaries were collected from Harbin XinCheng Food Co., Ltd. Porcine sperm was provided by the Northeast Agricultural University Embryo Engineering Laboratory Experimental Pig Base. ICR mice, for mouse embryonic fibroblast (MEF) cells, were purchased from Vital River Laboratories.

All animal care and investigations in this study were approved by the Ethics Committee of Northeast Agricultural University and complied with the Guide for the Care and Use of Laboratory Animals.

### *In vitro* oocyte maturation

Porcine ovaries were collected and kept in saline containing penicillin and streptomycin at 32–37°C. Antral follicles (2–8 mm in diameter) were aspirated using 10-gauge needles. High-quality oocytes with evenly granulated cytoplasm and at least three uniform layers of compact cumulus cells were selected and cultured in maturation medium (TCM-199 (Gibco) supplemented with 0.07 mg/ml cysteine (Sigma), 0.05 mg/ml epidermal growth factor (EGF, Sigma), 0.5 mg/ml luteinizing hormone (LH, Sigma), and 0.5 mg/ml follicle-stimulating hormone (FSH, Sigma)) at 39°C in a 5% CO_2_ atmosphere. At 42 hours after maturation, porcine oocytes with the first polar body were considered mature oocytes and were selected for further experiments.

### In vitro fertilization of porcine oocytes

Each group, containing 30 mature oocytes, was placed in a 50 μl droplet of modified Tris-buffered medium (mTBM) for fertilization. Fresh sperm kept at 17°C was briefly warmed at 39°C, and then washed two times in Dulbecco’s phosphate-buffered saline (DPBS, Sigma) supplemented with 0.1% (w/v) bovine serum albumin (BSA, Sigma) by centrifuging the sample at 2,000 rpm for 4 minutes. The suspended sperm was added into the droplets with oocytes at a final concentration of 3×10^5^/ml, and the mixtures were incubated at 39°C for 6 hours. Finally, the fertilized embryos were cultured in porcine zygote medium-3 (PZM-3) in 4-well plate at 39°C in a 5% CO_2_ atmosphere.

### Preparation of feeder cells

MEF cells were isolated from 13.5 days post-coitum ICR mouse foetuses. The minced tissues were digested with 1.6 mg/ml collagenase IV (Sigma) and 25 units of DNase I (Sigma) at 37°C, and then the dispersed cells were cultured in 15% FBS medium (high-glucose Dulbecco’s modified Eagle medium (DMEM, Gibco) with 15% foetal bovine serum (FBS, Bioind), 1% (v/v) nonessential amino acids (NEAA, Gibco), 0.29 mg/ml L-glutamine (Sigma) and 100 units/ml penicillin-streptomycin (Gibco)) at 37°C in a 5% CO_2_ atmosphere. At the third passage, the confluent cells were treated with 10 μg/ml mitomycin C for 2–3 hours and were stored in liquid nitrogen using a standard procedure[33].

### Isolation and culture of porcine pluripotent stem cells

Porcine blastocysts (>200 μm in diameter) produced *in vitro* were chosen at different days (5.5 days, 6.5 days and 7.5 days) to establish pluripotent cell lines. Zona pellucida were removed by mechanical dissection under the microscope using pulled glass pipettes, and then the whole, zona-free blastocysts were seeded on mitotically inactivated feeders in three types of media, namely, LN medium, BH medium and MXV medium. The LN medium included 48% DMEM/F12 (Gibco), 48% Neurobasal (Gibco), 100 units/ml penicillin-streptomycin, 0.5% N2 (Gibco), 1% B27 (Gibco), 0.25 mg/ml BSA, 0.29 mg/ml L-glutamine, 7.8 μg/ml β-mercaptoethanol (Gibco), 40 μg/ml vitamin C (Sigma) and 5 ng/ml human LIF (Millipore). The BH medium, commonly used as human ESC culture medium, contained 76% knockout Dulbecco's modified Eagle medium (KO-DMEM, Gibco), 20% knockout serum replacement (KOSR, Gibco), 0.145 mg/ml L-glutamine, 100 units/ml penicillin-streptomycin, 3.9 μg/ml β-mercaptoethanol, 0.5% NEAA and 8 ng/ml bFGF (R&D). The MXV medium, a newly developed culture medium that was modified from MX [34], consisted of 38% KO-DMEM, 24% DMEM/F12, 24% Neurobasal, 10% KOSR, 0.145 mg/ml L-glutamine, 100 units/ml penicillin-streptomycin, 3.9 μg/ml β-mercaptoethanol, 0.5% NEAA, 0.25% N2, 0.5% B27, 0.25 mg/ml BSA, 40 μg/ml vitamin C, 5 ng/ml human LIF and 8 ng/ml bFGF. After 5–7 days of culture, the fully expanded outgrowths were mechanically isolated from the feeder cells, dissociated into several clumps using pulled glass pipettes, and transferred onto new feeder cells for subculture. The media were changed every day, and the stable porcine pluripotent stem cell (pPSC) line could be passaged by 1 mg/ml collagenase IV every 5–7 days. The cells were cultured in humidified conditions with 5% O_2_, 5% CO_2_ and 90% N_2_ at 39°C.

### Cryopreservation and resuscitation

The pPSCs were dissociated into small clumps using 1 mg/ml collagenase IV, and then the cells were collected by centrifugation and suspended in 0.5 ml of freezing buffer (45% FBS, 45% KOSR and 10% DMSO (Sigma)) supplemented with 10 μmol/L Y27632, an inhibitor of Rho-associated protein kinases (EMD4, Biosciences). The cells were frozen at—80°C for 12 hours and were subsequently transferred into liquid nitrogen for storage. To recover the frozen cells, the sample tube was immediately placed in a 37°C water bath. The thawed cells were suspended with 5 ml of MXV medium and were centrifuged at 1200 rpm for 3 min. The pPSCs were plated onto fresh feeders with MXV medium containing 10 μmol/L Y27632.

### Alkaline phosphatase staining

The pPSCs were fixed with 4% (w/v) paraformaldehyde for 90 seconds at room temperature and were washed three times with DPBS. Alkaline phosphatase (AKP) staining was performed with a BCIP/NBT Alkaline Phosphatase Colour Development Kit (Beyotime) following the manufacturer’s instructions. The cells were examined using an inverted microscope.

### Karyotype analysis

The pPSCs were exposed to 0.4 μg/ml colchicine (Sigma) for 24 hours and were dissociated into single cells using 0.25% (w/v) trypsin-EDTA. Then, the cells were treated with KCl solution (0.075 mol/L) at 37°C for 15 min. Fixation was performed with methanol:glacial acetic acid (3:1) at 4°C for 40 min, and the cells were spread onto ice-cold slides. After being allowed to dry for 5 min, the spreads were stained with Giemsa and were observed using a Nikon 80i microscope. Thirty samples were selected for observation and chromosome number counting.

### Embryoid body (EB) formation and spontaneous differentiation

The pPSCs were treated with 1 mg/ml collagenase IV, cultured in non-coated plastic dishes with MXV medium without bFGF and LIF for 1 day, and then transferred to 15% FBS medium for another 5–7 days. Typical EBs were transferred to 0.1% (w/v) gelatine-coated dishes and were cultured in 15% FBS medium for 4 additional weeks. The medium was changed every other day. Morphologic observation and immunofluorescence were used to evaluate the differentiation of the three germ layers.

### Immunofluorescence analysis

The pPSCs and the differentiated cells were fixed with 4% (w/v) paraformaldehyde (PFA) for 40 min at room temperature and were washed three times with phosphate-buffered saline (PBS, Sigma). Then, they were permeabilized with 1% (v/v) Triton X-100 in PBS overnight at 4°C and were blocked with 1% (w/v) BSA in PBS for 1 hour at 37°C. They were incubated with primary antibodies for OCT4 (Santa Cruz, Sc-8628, 1:50), SOX2 (Santa Cruz, sc-17320, 1:250), NANOG (Pepro Tech, 500-P236, 1:250), CDX2 (Biogenex, MU392A-UC, 1:50), AFP (Abnova, H00000174, 1:50), VIMENTIN (Sigma-Aldrich, V6630, 1:50) and β-TUBULIN (Sigma-Aldrich, T5201, 1:100) overnight at 4°C. After they were incubated, the cells were washed three times with 0.01% (v/v) Triton X-100 and 0.1% (v/v) Tween-20 in PBS at room temperature. The cells were then incubated with secondary antibodies (Alexa Fluor® 488 for donkey anti-goat, donkey anti-rabbit and donkey-anti mouse, Invitrogen) diluted at 1:500 in 0.01% (v/v) Triton X-100 and 0.1% (v/v) Tween-20 in PBS for 1 hour at 37°C. Then, the cells were washed three times. Cell nuclei were stained with Hoechst 33342 (Sigma) for 8 minutes and were subsequently washed three times. Finally, the stained cells were mounted on glass slides and examined using a Nikon 80i microscope.

### Teratoma formation

Before the treatment, the pPSCs were incubated in medium with 10 μmol/L Y27632 for at least 2 hours. Then, the cells were dissociated into small clumps by 1 mg/ml collagenase IV and were centrifuged at 1200 rpm for 3 min. The cells were suspended with DPBS containing 10 μmol/L Y27632. The pPSC cells (5×10^7^) were injected subcutaneously into 8-week-old nude mice. After two months, the teratomas were collected, dissected and fixed in 4% (w/v) PFA, processed for paraffin section, and stained with haematoxylin and eosin (H&E).

### Lentivirus production and gene transduction

Lentiviral plasmids containing red fluorescent protein (DsRed) and the packaging plasmids (PSPAX and PMD.2G) were purchased from Addgene. 293T cells were transfected with 24 μg of plasmids, 48 μl of LTX and 24 μl of PLUS regents, and the proportions of PMD.2G, PSPAX and DsRed were 1:2:3. The supernatants were collected at 24 h and 48 h after transfection, filtered by 0.45 μm filters, and concentrated by centrifugal filters (Millipore) at 4°C and 4000 g for 30 minutes. The pPSCs (>10 passages) were transduced with concentrative lentivirus with a multiplicity of infection (MOIs) of 5 for 24 hours in MXV medium containing 8 mg/ml polybrene. To obtain a purified population of DsRed-expressing cells, positive cells were mechanically isolated from the others under 510–560 nm excitation wavelength light (G2A light), dissociated into several clumps using pulled glass pipettes, and transferred onto new feeder cells for subculture. This process was repeated every 5 passages.

### Somatic cell nuclear transfer (SCNT)

Donor cells were injected into the perivitelline space of enucleated porcine oocytes with a glass pipette 25 μm in diameter, and the electro-fusion parameters were two direct pulses of 120 V/mm for 30 msec. The fused eggs were cultured in PZM-3 medium for 7 days at 39°C in a 5% CO_2_ atmosphere. The cleavage and blastocyst rates were assessed at 48 hours and 168 hours after activation. Three types of donor cells were employed for SCNT, including pPSCs, DsRed-marked pPSCs (pPSC-FDs) and DsRed-marked porcine embryonic fibroblasts (PEF-FDs).

### Generation of Chimaeras

Before cell dissociation, pPSC-FDs were incubated in medium with 10 μmol/L Y27632 for at least 2 hours. Then, the cells were mechanically isolated from the feeder cells and were dissociated into small clumps (2–3 cells) by TrypLE digestion drops (Gibco). Twelve pPSC-FDs were injected gently into the cavity of day 4.5 blastocysts using a piezo-assisted micro-pipette. The injected blastocysts were cultured in PZM-3 at 39°C in a 5% CO_2_ atmosphere for 24 hours, and then day 5.5 chimeric blastocysts were surgically transferred to recipient sows on day 6 of pseudopregnancy. Foetuses were obtained after 50 days of gestation, and chimaeras were confirmed by the expression of DsRed fluorescent protein in foetuses or placentas.

### RT-PCR and quantitative PCR

Total RNA was extracted from the collected samples using an RNeasy mini kit (Qiagen) according to the manufacturer’s instructions. Reverse transcription was performed using High-Capacity cDNA Reverse Transcription Kits (Applied Biosystems, 4368814) according to the manufacturer’s instructions. Quantitative real-time PCR was performed using Premix Ex TaqTM (Perfect Real Time, TaKaRa) and a 7500 Real-Time PCR System (Applied Biosystems). All the primers are listed in Table 1. PEFs and porcine induced pluripotent stem cells (piPSCs, a pluripotent stem cell line derived from our own lab) were used as controls [34].

**Table 1:** Primer sequences for PCR.

Gene name	Forward primers	Reverse primers
*OCT4*	CAAACTGAGGTGCCTGCCCTTC	ATTGAACTTCACCTTCCCTCCAACC
*SOX2*	CATCAACGGTACACTGCCTCTC	ACTCTCCTCCCATTTCCCTCTTT
*NANOG*	CCTCCATGGATCTGCTTATTC	CATCTGCTGGAGGCTGAGGT
*LIF*	CACTGGAAACACGGGGCA	AGGGCGGGAAGTTGGTCA
*LIFR*	CTCATCCCAGTGGCAGTG	CCAGAACCTCAACATTAT
*BMP4*	AGCATGTCAGGATTAGCCGA	TGGAGATGGCACTCAGTTCA
*bFGF*	GCGACCCTCACATCAAACT	CAGTGCCACATACCAACT
*FGFR1*	ACTGCTGGAGTTAATACCACCG	GCAGAGTGATGGGAGAGTCC
*FGFR2*	TGATGATGAGAGACTGTTGGCATGC	TCCAAGTAGTCCTCATTGGTCGTG
*SMAD4*	GGCTTCAGGTGGCTGGTCGGA	ACCTGATGGAGCATTACT
*CDX2*	GCTATAAATGCCAGAGCCAACC	AACAACCCAAACAGCAGCAAC
*TEAD4*	AAGGCCGGCACCATTACCT	CAGCTCATTCCGACCGTACAT
*Gata3*	TCCTACTACGGAAACTCGGTGAGG	CGTCTTGGAGAACGGGCTGAGGTT
*GAPDH*	GCAAAGTGGACATTGTCGCCATCA	TCCTGGAAGATGGTGATGGCCTTT
*beta-ACTIN*	AGATCGTGCGGGACATCAAG	GCGGCAGTGGCCATCTC

### Statistical analysis

Statistical analysis was performed using SPSS 19.0. The data are presented as the mean ± S.D. One-way analysis of variance (ANOVA) was used to assess any differences between groups. P<0.05 was considered statistically significant.

## Results

### Generation of a porcine pluripotent stem cell (pPSC) line from IVF blastocysts

**Fig 1:**
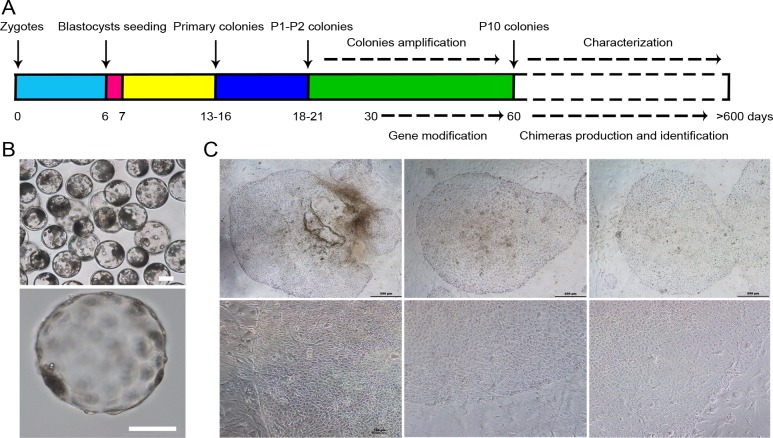
Generation of pPSCs from porcine pre-implantation embryos. (A) Strategy to establish and identify the pPSCs. (B) Day 5.5 porcine blastocysts fertilized *in vitro* and chosen for seeding. The upper image represents the porcine blastocysts fertilized *in vitro*. The bottom image is a zona pellucida-free blastocyst prior to being seeded. Scale bars = 100 μm. (C) Morphology of pPSCs. Scale bars = 500 μm (first row) and 100 μm (second row).

It has been reported that physiological O_2_ conditions (4%-5% O_2_) could favour glycolytic metabolism in human embryonic stem cells [35], control human ESCs in an undifferentiated state [36], and enhance the reprogramming efficiency [37] and maintain the active chromatin state of pluripotent cells[38]. For these reasons, we chose 5% O_2_ concentration as the basic gas culture condition in this study. Next, we compared the effects of different culture media on the derivation of outgrowths from day 6.5 IVF porcine blastocysts to select an optimal culture condition for porcine ESCs. As shown in Table 2, more outgrowths (8.8%) were obtained when we seeded the whole, zona-free blastocysts in the MXV medium, in which the derivation efficiency was higher than those in other groups. Therefore, we used MXV medium to determine the optimal development stage of pre-implantation embryos selected for pPSC derivation as well as to perform stable passages. As summarized in Table 3, there were no significant differences in the percentages of attachment among the blastocysts of different stages. However, the percentage of outgrowth (17.18%) and stably passaged cell lines (74.33%) in day 5.5 blastocysts were significantly higher than those of day 6.5 and day 7.5 blastocysts. According these results, we established an optimized isolation and culture system for pPSCs (Fig 1A). In this system, zona-free, day 5.5 (≥200 μm in diameter) blastocysts were selected to be seeded on feeder cells (Fig 1B). The attached blastocysts were cultured in MXV medium and humidified conditions with 5% O_2_, 5% CO_2_ and 90% N_2_ at 39°C, and the outgrowths formed on the seventh day after seeding (Fig 1C). The fully expanded colonies were mechanically isolated from the feeder cells, dissociated into several clumps using pulled glass pipettes, and plated onto new feeder cells for subculture. The stably passaged cells were named porcine pluripotent stem cells (pPSCs), and one of the cell lines has been cultured for more than two years (>75 passages); these cells could be passaged by 1 mg/ml collagenase IV every five to seven days. The pPSC colonies were flat in morphology, similar to those of human ESCs (Fig 1C).

**Table 2:** The effects of media on the establishment of porcine pluripotent stem cell lines derived from day 6.5 blastocysts.

Media	Embryos	Attached embryos (%)	Outgrowth (%)	Stable passage (%)
BH	49	39 (80.57±4.88)^a^	0^a^	0
LBN	50	41 (83.19±15.46)^a^	1 (1.96±3.46)^a^	0
MXV	41	34 (84.64±14.55)^a^	4 (8.80±2.64)^b^	0

The experiment was repeated three times.

^a^ Values with different superscript symbols in the same column differ significantly (P<0.05).

^b^ Values with different superscript symbols in the same column differ significantly (P<0.05).

**Table 3:** The effect of embryonic age on the establishment of porcine pluripotent stem cell lines cultured in 5% oxygen concentration and MXV medium.

Embryonic age (Days)	Embryos	Attached embryos (%)	Outgrowth (%)	Stable passage (%)
5.5	163	147(89.81±3.61)^a^	23(17.18±9.54)^a^	17 (74.33±4.04)^a^
6.5	183	160(87.67±3.15)^a^	2(1.33±1.16)^b^	0^b^
7.5	159	134(82.25±8.54)^a^	1(1.10±1.73)^b^	0^b^

The experiment was repeated three times.

^a^ Values with different superscript symbols in the same column differ significantly (P<0.05).

^b^ Values with different superscript symbols in the same column differ significantly (P<0.05).

### Characterization of pPSCs

**Fig 2:**
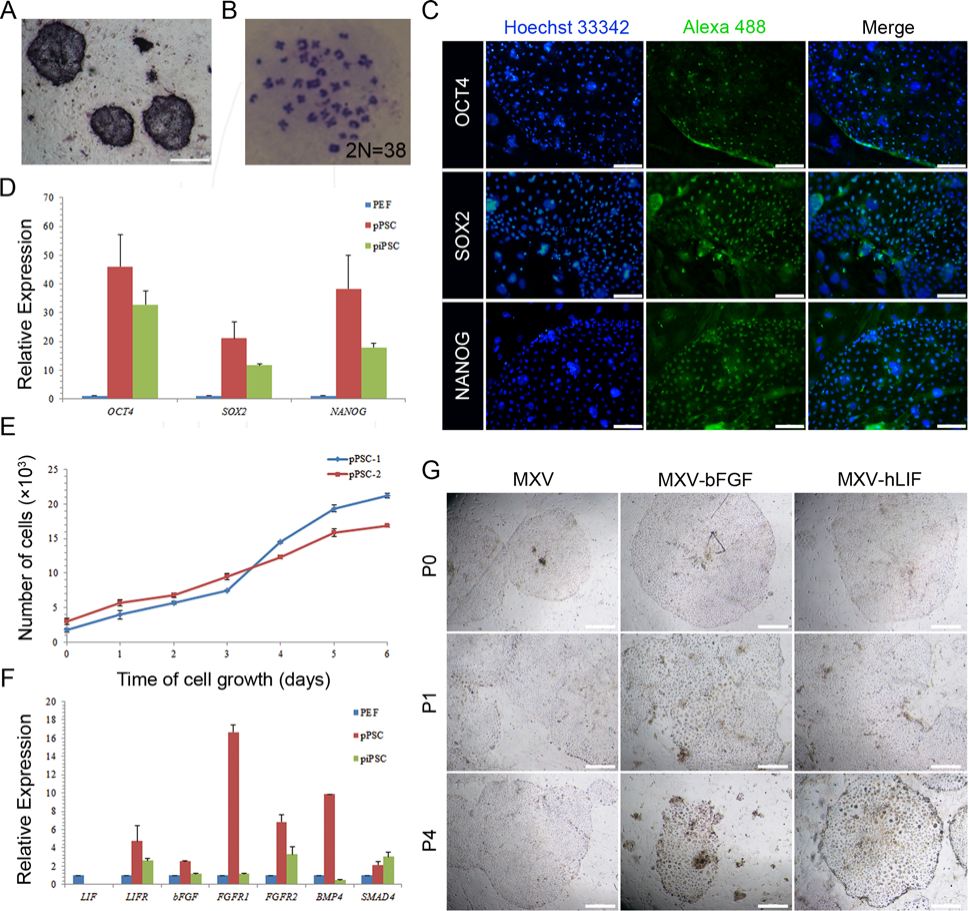
Characterization of pPSCs. (A) pPSC colonies showed positive staining for alkaline phosphatase. Scale bars = 500 μm. (B) Karyotype analyses of pPSCs showed normal porcine karyotype of 38 chromosomes. (C) Immunostaining for pluripotent markers of pPSCs. The colonies showed positive staining for OCT4, SOX2 and NANOG. Nuclei were stained with Hoechst 33342. Scale bars = 100 μm. (D) Quantitative RT-PCR for *OCT4*, *SOX2* and *NANOG*. The expression levels of these genes were relative to the expression of *GAPDH*. (E) Proliferation detection. Doubling time: TD = t[lg2/(lgNt-lgNo)] = 45.5 h. (F) Quantitative RT-PCR for *LIF*, *LIFR*, *bFGF*, *FGFR1*, *FGFR2*, *BMP4*, and *SMAD4*. The expression levels of these genes were relative to the expression of *GAPDH*. (G) Morphology of pPSCs of different passages in the MXV medium without bFGF or LIF. Scale bars = 500 μm.

To describe the characterization of the established pPSCs, we performed a series of pluripotent identifications. The results indicated that the pPSCs were alkaline phosphatase positive with a normal karyotype of 38 chromosomes (Fig 2A and 2B). The immunofluorescence assay demonstrated that the pPSCs were positive for pluripotent markers OCT4, SOX2, and NANOG, but not CDX2, a typical marker for trophectoderm (Fig 2C and S1 Fig). We also characterized the pluripotent markers using quantitative RT-PCR, and the results confirmed that the classic pluripotent markers, including *OCT4*, *SOX2* and *NANOG*, were remarkably expressed in the pPSCs (Fig 2D). Then, we assayed the proliferation potential of the pPSCs by measuring the cumulative cell increase. The results indicated that the pPSCs grew at a doubling time of 45.5 hours, which was similar to that of human ESCs (Fig 2E).

### The pluripotency of pPSCs depends on FGF and LIF signalling pathways

It has been shown that the self-renewal and pluripotency of human ESCs and mouse ESCs rely on the FGF and LIF signalling pathways, respectively. To identify the pathway required to sustain undifferentiated pPSCs, we examined key downstream effector molecules of the FGF and LIF pathways using quantitative RT-PCR. The results showed that the pPSCs expressed *FGFR1*, *FGFR2*, *LIFR* and *BMP4* (Fig 2F), which indicated that the pPSCs might rely on both the FGF pathway and the LIF pathway to maintain proliferation and pluripotency. To further test this, we cultured pPSCs in MXV medium without bFGF or LIF. As shown in Fig 2G, the cells could not maintain typical clonal morphology in the medium without bFGF; in addition, the cells would not proliferate well, and colonies became loose when LIF was withdrawn. These results confirmed that the pluripotency of our pPSCs depended on both the FGF and LIF signalling pathways.

### *In vitro* and *in vivo* differentiation of pPSCs

**Fig 3:**
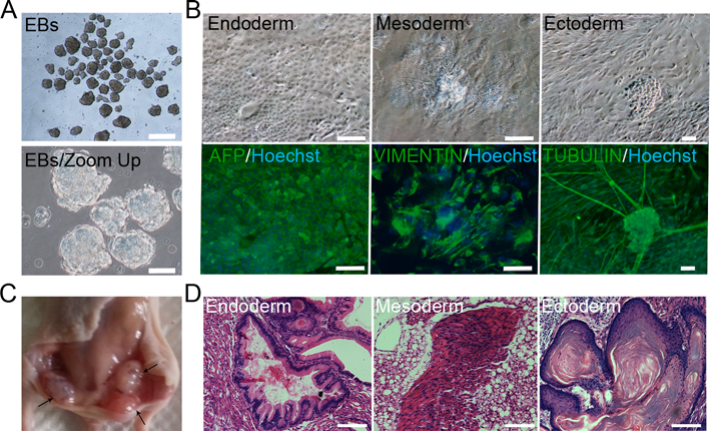
*In vitro* and *in vivo* differentiation of pPSCs. (A) *In vitro* embryoid body (EB) formation and *in vitro* differentiation. Scale bars = 500 μm (top) and 100 μm (bottom). (B) EBs spontaneously differentiated into cells of the three germ layers. The differentiated cells showed positive staining for alpha fetoprotein (AFP, endoderm marker), VIMENTIN (mesoderm marker) and beta-TUBULIN (ectoderm marker). Nuclei were stained with Hoechst 33342 (blue). Scale bars = 500 μm (AFP and VIMENTIN) and 100 μm (TUBULIN). (C) Teratomas derived from pPSCs. (D) Histological examination pPSCs showed three germ layer differentiations of pPSCs in teratomas. Scale bars = 100 μm.

To validate the pluripotency of the pPSCs, we analysed the differentiated abilities of the cells *in vitro* and *in vivo*. The pPSCs could form embryoid bodies (EBs) in non-coated plastic dishes at different generations (Fig 3A). When grown in gelatine-coated dishes, the EBs spontaneously differentiated into cell types of the three germ layers, as determined by the immunofluorescence assay and morphological observation after 4 weeks. The detected cells were positive for AFP (alpha-fetoprotein, endoderm marker), VIMENTIN (mesoderm marker), and β-TUBULIN (ectoderm marker) (Fig 3B). For *in vivo* differentiation, the pPSC cells were subcutaneously injected into nude mice. Teratomas were obtained as isolated masses (Fig 3C), and histological examination revealed that the teratomas contained classic tissues of the three germ layers, including digestive tube (endoderm), muscle (mesoderm), and skin (ectoderm) tissue (Fig 3D).

### Exogenous DsRed could be successfully transduced into pPSCs

**Fig 4:**
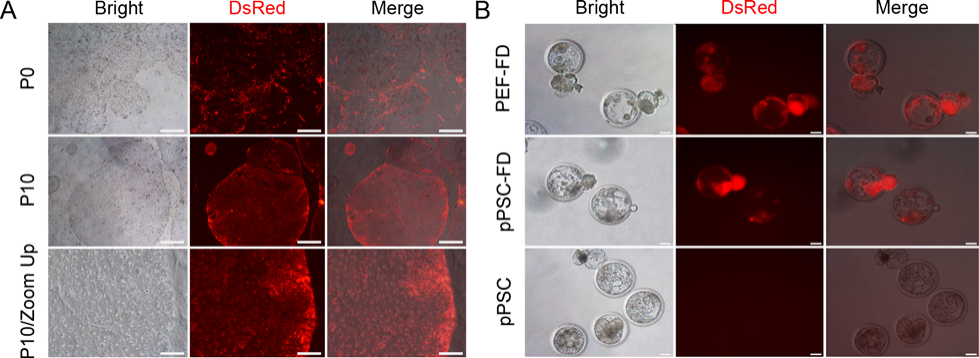
pPSCs transfected with fuw-DsRed (pPSC-FD) showed stable pluripotency. (A) pPSCs transfected with fuw-DsRed showed stable transgene expression during passaging. Scale bars = 500 μm (P0 and P10) and 100 μm (inset). (B) Day 7 NT embryos derived from pPSC-FDs developed into blastocysts with strong DsRed expression. Embryos derived from fuw-DsRed-transfected PEFs (PEF-FDs) and pPSCs served as controls. Scale bars = 100 μm.

Furthermore, red fluorescent protein (fuw-DsRed) was successfully introduced into the pPSCs via lentiviral vectors. After continuous selection and purification, all the cells expressed DsRed with unimpaired pluripotency (Fig 4A and S2 Fig). When the transgenic cells were used for somatic cell nuclear transfer (SCNT), 11.52% of NT embryos could develop into blastocysts (Fig 4B and Table 4), which was not significantly different from that of the reconstructed embryos derived from porcine embryonic fibroblasts.

**Table 4:** The developmental potency of SCNT embryos using porcine pluripotent stem cells as donor cells.

Groups	Embryos	Fusion (%)	Cleavage (%)	Blastocyst (%)
pPSC	330	212 (64.86±9.85)^a^	123 (58.33±3.51)^a^	36 (17.15±2.00)^a^
pPSC-FD	264	180 (69.67±7.59)^a^	121 (60.21±10.54)^a^^b^	20 (11.52±2.31)^b^
PEF-FD	182	100 (54.33±8,51)^a^	74 (76.33±10.02)^b^	14 (13.67±2.52)^a^^b^

The experiment was repeated three times.

^a^ Values with different superscript symbols in the same column differ significantly (P<0.05).

^b^ Values with different superscript symbols in the same column differ significantly (P<0.05).

### Chimeric viscera and placentas produced from fuw-DsRed transfected pPSCs (pPSC-FDs)

**Fig 5:**
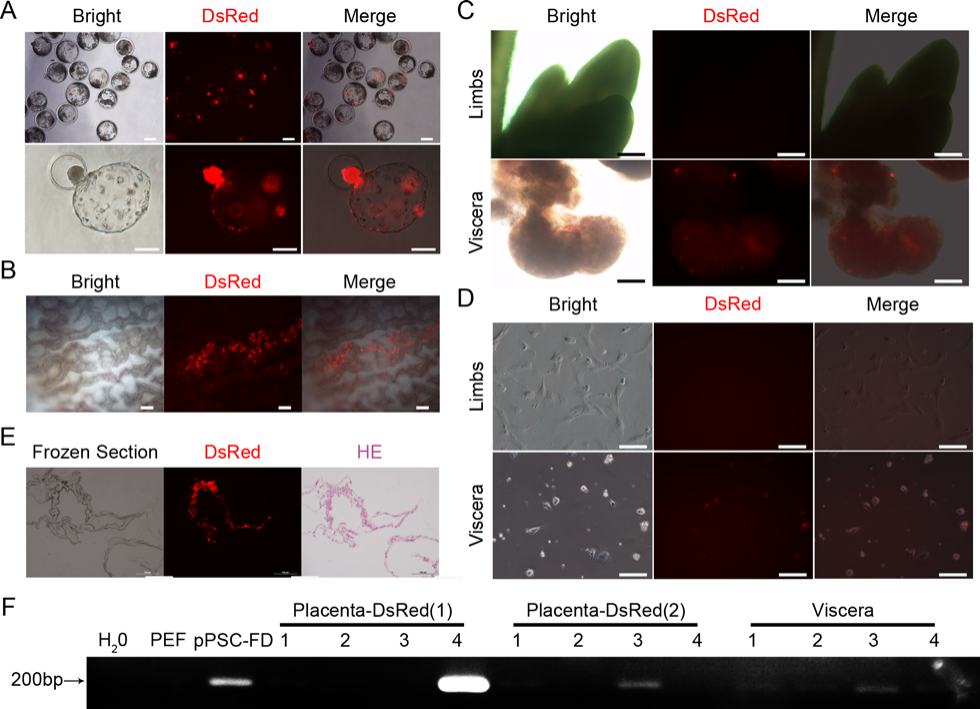
pPSC-FDs could contribute to placental chimaera. (A) Generation of chimeric blastocysts when pPSC-FDs were injected into the small cavity of IVF blastocysts. Scale bars = 100 μm. (B) Detecting the chimaerism in the day 50 placentas by direct observation under G2A light. The fluorescent cells in the placentas indicated the contribution of the pPSC-FDs. Scale bars = 100 μm. (C) Assessing the chimeric development of the limbs and viscera in the day 50 foetuses by direct observation under G2A light. Scale bars = 500 μm. (D) Tracking the DsRed-positive cells in the cultured limbs and viscera under G2A light. Cells derived from the viscera showed red fluorescence, which indicated a pPSC-FD contribution in the viscera. Scale bars = 500 μm. (E) Examining the chimeric development of placental tissues using cryosections. The sections were stained by haematoxylin and eosin. Scale bars = 100 μm. (F) PCR showed fuw-DsRed expression in the chimeric placentas and viscera. DsRed (1) and DsRed (2) represent two different parts of the same placenta. The numbers 1, 2, 3 and 4 refer to the four foetuses.

To further investigate the developmental potential of the pPSCs, fuw-DsRed-transfected pPSCs (pPSC-FDs) were used to construct chimeric embryos. The pPSC-FDs were injected into the cavity of day 4.5 porcine blastocysts fertilized *in vitro*, and they participated in the embryonic development, as indicated by the red fluorescence detected after another 2.5 days of incubation *in vitro* (Fig 5A). Subsequently, 354 chimeric blastocysts were transferred into 8 recipient sows that had undergone oestrus synchronization. At 50 days after embryo transplantation, 4 foetuses were collected from a pregnant sow. Foetuses and placentas were examined for the chimaerism separately. For two of the four foetuses, red fluorescent signals could be distinctly observed in the corresponding placentas under 510–560 nm excitation wavelength light (G2A light, Fig 5B), but the signals were weak in the viscera and non-existent in the limbs (Fig 5C). To confirm the chimerism, the limbs and viscera were dissected into pieces and cultured for further observation, and red cells were found in the viscera (Fig 5D). DsRed-positive chimeric placentas were used to prepare cryosections, and the slices were stained using haematoxylin and eosin. pPSC-FDs were observed in the placenta tissue (Fig 5E), which was confirmed by the PCR results (Fig 5F). To our knowledge, these pPSCs are the first porcine pluripotent cells that could contribute to both the viscera and extraembryonic tissues.

## Discussion

In this study, we derived a porcine pluripotent stem cell (pPSC) line from day 5.5 *in vitro fertilization* blastocysts in a newly developed condition. The pPSCs had some pluripotent properties in common with those of mouse ESCs and human ESCs, such as expressing AKP, OCT4, SOX2 and NANOG, showing a flat morphology, and maintaining pluripotency through more than 75 passages over two years. Under appropriate differentiation conditions, the pPSCs could differentiate *in vitro* into cells of the three germ layers through embryoid bodies and could form teratomas in nude mice that included derivatives of tissues from the three germ layers. The pPSCs could also harbour exogenous DsRed in their genome without losing their pluripotency and passaging stability. Notably, the pPSCs had the ability to integrate into both ICM cells and trophoblast cells in the chimeric blastocysts when the labelled cells were injected into day 4.5 early blastocysts, and the cells were able to contribute to the chimaera development when the embryos injected with pPSCs were transferred to recipients. In conclusion, all of the characterization techniques indicated that the pPSCs derived in this study have formed an attractive and valuable porcine pluripotent stem cell line that has not previously been reported.

Porcine ESC-like cells have been derived from early embryos in past decades, but either the passaging capability or the differentiation potency was unsatisfactory, which limited further practical applications in genetic engineering and in studies of porcine-specific molecular mechanisms of pluripotency regulation. Yang et al. reported the longest-passaged porcine ESC-like cell line, which was derived from ICM of day 7 *in vivo* blastocysts and could be passaged *in vitro* for more than 7 years[39]. Although the cell line showed a flat colony morphology that was similar to that of human ESCs, and the cells expressed pluripotent markers such as AKP, OCT4, SSEA4, TRA-1-60 and TRA-1-81, it could not form teratomas in severe combined immune deficiency (SCID) mice large enough to demonstrate the differentiation of tissues from the three germ layers. There have been a few reports on the formation of teratomas [7, 11, 18, 25, 40], which claimed that teratomas could be obtained from cell lines derived from day 10–12 embryos[11, 18], but not from day 5–6 or day 7–8 embryos[6, 18]. However, all of these cell lines were derived from *in vivo* embryos and exhibited limited proliferation capability *in vitr*o. Recently, Jung et al. stated that they established a parthenogenetic embryonic stem cell line from day 7 parthenogenetic embryos[25]. In contrast with these cell lines, the pPSCs established in this study showed great advances in passaging capability and differentiation.

There are two pluripotent states represented by mouse ESCs and human ESCs, which are called naïve and primed pluripotency, respectively. Although they share common pluripotent properties, such as the expression of the core transcription factors OCT4, SOX2 and NANOG, unlimited proliferation *in vitro* with a normal karyotype, and differentiation of the three germ layers *in vitro* and *in vivo*, the differences between them are obvious. Except for the compact dome morphology in mouse ESCs and the loose, flat morphology in human ESCs, the self-renewal and proliferation of mouse ESCs depends on LIF/STAT3 signalling, but that of human ESCs depends on FGF and TGF-beta signalling. Female mouse ESCs show an absence of X inactivation, but human ESCs show random X inactivation [41–43]. Studies of porcine pluripotent cells, including porcine ESC-like cells and porcine induced pluripotent stem cells (piPSCs), have shown that the signalling pathway involved in sustaining the pluripotency of the porcine cells was underdetermined. Brevini et al. and Blomberg et al. reported that they detected the expression of *STAT3* in their cell lines, but the absence of *GP130* and *LIFR*[20, 44]. Several other researchers have demonstrated that LIF was indispensable for maintaining the proliferation of porcine ESC-like cells [22, 40, 45]. Recent results have tended to show that both FGF and LIF signalling are necessary for porcine pluripotency [25, 32], especially the results of a study of piPSCs with mouse ESC morphology [34]. The pPSCs derived in this study are FGF and LIF dependent, as they could not maintain the typical clonal morphology without bFGF and grew slowly without LIF. These results indicated that the porcine pluripotency regulation networks might be different than those of mouse ESCs and human ESCs. This species-specific property has also been demonstrated in the regulation of non-human primate naïve pluripotency [46].

Stem cell genetic engineering offers powerful tools for functional genomics and disease modelling. It has been demonstrated that transferring exogenous genes into porcine ESC-like cells was inefficient, and the genes were not stably expressed[47]. There have been several other reports on the transgenic manipulation of porcine pluripotent cells; Yang et al. established hrGFP transgenic porcine ESC-like cells by electroporation[39], and Rui et al. reported eGFP transgenic porcine EG-like cells by liposomal transformation[48]. The transgenic hrGFP porcine ESC-like cells showed a steady expression of exogenous genes and pluripotent markers, but they did not form teratomas with the differentiation of the three germ layers [39]. The transgenic eGFP porcine EG-like cells were integrated into the development of blastocysts *in vitro*[48], but there was no description on the chimerism after further development *in vivo*. The pPSCs in this study were transfected using a lentiviral vector and expressed DsRed stably while being passaged, and they maintained the pluripotent properties. The transgenic DsRed pPSCs (pPSC-FDs) could support reconstructed embryos developing into blastocysts when they were used as nuclear donors, could contribute to the development of both ICM cells and trophoblast cells *in vitro* when they were injected into blastocysts, and could contribute to the development of the viscera and placenta *in vivo* when the chimeric blastocyst were transferred into recipients. These results indicated that this cell line could have practical significance on future research of porcine pluripotency as well as human disease modelling.

It is impressive that the pPSCs in this study could contribute to both ICM cells and trophectoderm at the blastocyst stage, as well as contribute to the development of the viscera and placenta *in vivo*. Although Vassiliev et al. indicated that their ESC-like cells could only become incorporated in ICM cells when injected into host blastocysts [49], Chen et al. found that their ESC-like cells could contribute to both ICM cells and trophectoderm [12]. The ability to become incorporated into the development of trophectoderm and placenta indicates that the identity of porcine stem cells derived from pre-implantation embryos are different from corresponding murine cells, although they might have a number pluripotent characteristics in common. This type of difference also exists in the pre-implantation embryos. OCT4 was expressed in both ICM cells and trophectoderm in early porcine blastocysts, while only in ICM cells in mouse blastocysts [50, 51]. In addition, the ability of murine and porcine cells to differentiate into ICM cells and trophectoderm of early blastocysts were different [52, 53]. Therefore, the differentiation potential of genuine porcine ESCs still needs to be defined.

The capability of contributing to the germ line in chimaeras is the most convincing criterion for naïve pluripotent stem cells. To date, there have been no reports on porcine embryo-derived stem cells that could contribute to the germ line, although it has been demonstrated that freshly isolated ICM cells are germline competent. [11, 54–56]. Chimeric piglets derived from porcine ESC-like cells have only been reported by Chen et al. [12] and Vassiliev et al. [49], and the chimerism was demonstrated by skin pigmentation and microsatellite analysis. It was difficult to determine the extent of the chimerism in either of the studies because there was no unique genetic marker, such as DsRed. The factor that impeded the genetic modification of these cells might be that the cells used for chimaera production were chosen from an early passage. In the present study, we did not obtain the birth of chimeric piglets due to the assessment the foetus chimerism at day 50 of pregnancy, but the pPSCs clearly showed a contribution to the placenta, which was indicated by the transfected DsRed. The ability of the pPSCs to be transfected with DsRed and passaged without losing pluripotency is a great advantage for tracing the developmental capabilities of the pPSCs, and it will benefit the application of the pPSCs in potential animal model testing of therapeutic cell transplantation.

In general, we established a porcine pluripotent stem cell line, which shares common properties of pluripotency with mouse ESCs and human ESCs. In addition, this cell line showed specific characteristics of pluripotent signalling dependency. The capabilities of these cells to undergo unlimited passaging, differentiate into three germ layers *in vitro* and *in vivo*, and contribute to the chimerism indicated that this cell line is valuable for basic research and can be practically used for human disease modelling.

## Supporting Information

S1 Fig Characterization of pPSCs for trophoblast stem (TS) cell-related markers. (A) Immunofluorescent staining against CDX2, a typical TS cell marker. The colonies showed negative staining of CDX2. Nuclei were stained with Hoechst 33342. Scale bars = 500 μm. (B) Quantitative RT-PCR analysis of TS cell markers in pPSCs. The expression levels of *CDX2*, *TEAD4* and *GATA3* were relative to the expression of *GAPDH*.
(TIF)

S2 Fig Pluripotent detection of pPSC-FDs. (A) pPSC-FDs were positively stained for alkaline phosphatase. Scale bars = 100 μm. (B) Karyotype analyses of pPSC-FDs showed the normal porcine karyotype of 38 chromosomes. (C) Quantitative RT-PCR analysis of pluripotent markers in pPSC-FDs. The expression levels of *OCT4*, *SOX2* and *NANOG* were relative to the expression of *beta-ACTIN*.
(TIF)
