# Supplementary figures and images for: Porcine Pluripotent Stem Cells Derived from IVF Embryos Contribute to Chimeric Development In Vivo
Source: PLoS One. 2016 Mar 18;11(3):e0151737. doi: 10.1371/journal.pone.0151737 (PMC4798268; doi:10.1371/journal.pone.0151737)

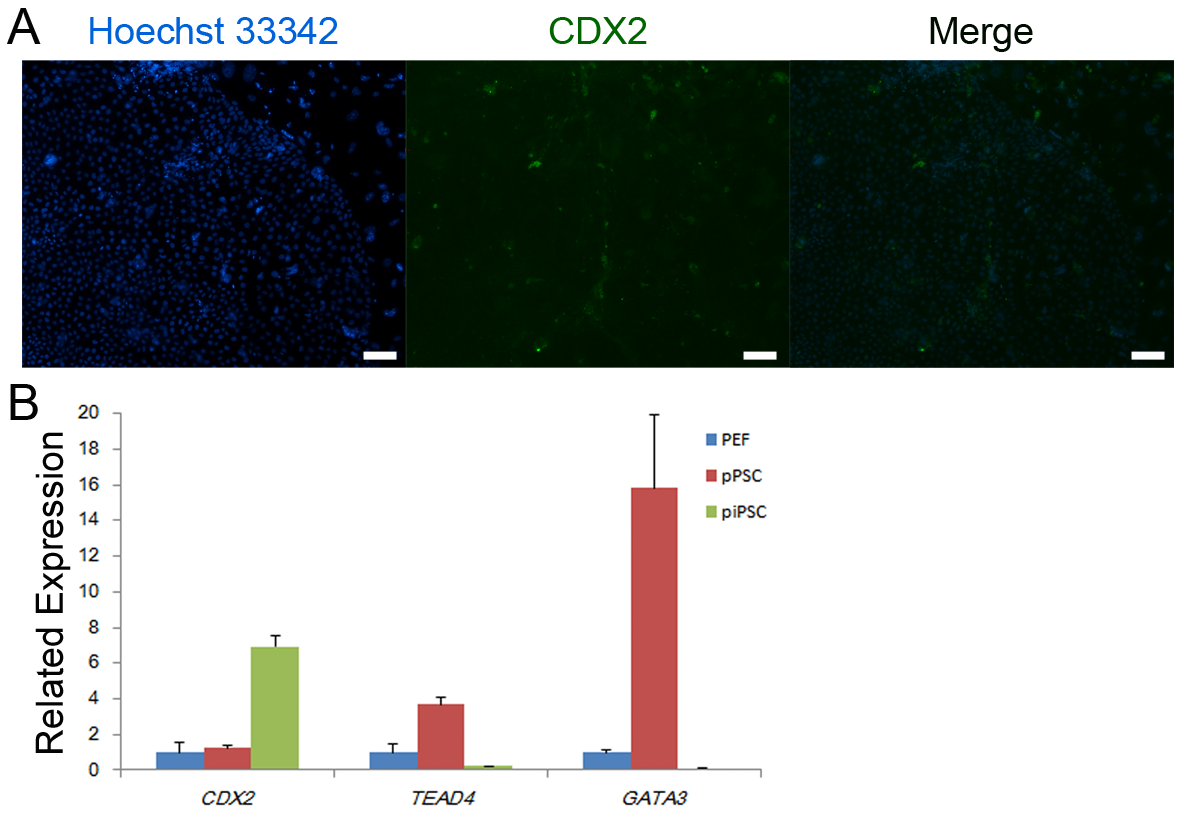

Supplement: S1 Fig — (A) Immunofluorescent staining against CDX2, a typical TS cell marker. The colonies showed negative staining of CDX2. Nuclei were stained with Hoechst 33342. Scale bars = 500 μm. (B) Quantitative RT-PCR analysis of TS cell markers in pPSCs. The expression levels of CDX2, TEAD4 and GATA3 were relative to the expression of GAPDH. (TIF) [file pone.0151737.s001.tif]

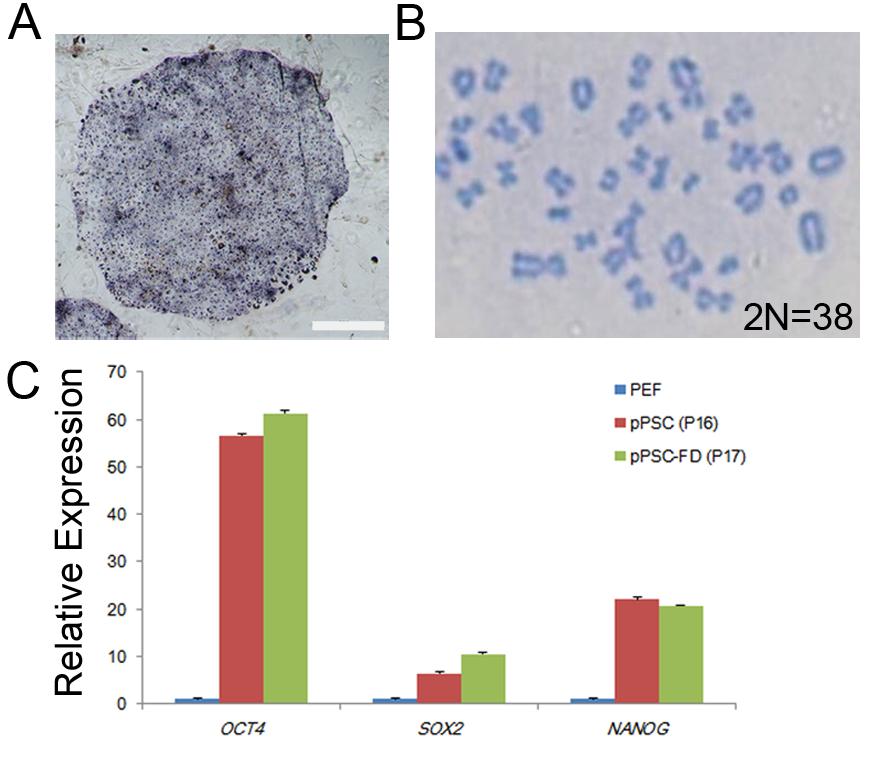

Supplement: S2 Fig — (A) pPSC-FDs were positively stained for alkaline phosphatase. Scale bars = 100 μm. (B) Karyotype analyses of pPSC-FDs showed the normal porcine karyotype of 38 chromosomes. (C) Quantitative RT-PCR analysis of pluripotent markers in pPSC-FDs. The expression levels of OCT4, SOX2 and NANOG were relative to the expression of beta-ACTIN. (TIF) [file pone.0151737.s002.tif]
